# Supplementary material for: Box–Behnken Design (BBD)-Based Optimization of Microwave-Assisted Extraction of Parthenolide from the Stems of Tarconanthus camphoratus and Cytotoxic Analysis
Source: Molecules. 2021 Mar 26;26(7):1876. doi: 10.3390/molecules26071876 (PMC8038025; doi:10.3390/molecules26071876)
Supplement: Supplementary file 1 [file molecules-26-01876-s001.pdf]

molecules

MDPI

Supplementary Material

Box–Behnken Design (BBD)-based optimization of microwave-assisted extraction of parthenolide from the stems of *Tarconanthus camphoratus* and cytotoxic analysis

Perwez Alam[1*], Nasir Ali Siddiqui[1], Md. Tabish Rehman[1], Afzal Hussain[1], Ali Akhtar[2], Showkat R. Mir[3], Mohamed Fahad Alajmi[1*]

[1]Department of Pharmacognosy, College of Pharmacy, King Saud University, Riyadh, KSA. Email: PA, aperwez@ksu.edu.sa ; NAS, nsiddiqui@ksu.edu.sa ; MTR, mrehman@ksu.edu.sa ; AH, afihussain@ksu.edu.sa ; MFA, malajmii@ksu.edu.sa
[2]IT & Quality Unit, College of Pharmacy, King Saud University, Riyadh, KSA. Email: AA, aakhtar@ksu.edu.sa
[3]Phyto-pharmaceutical Research Lab., School of Pharmaceutical Education and Research, Jamia Hamdard, New Delhi, India. Email: SRM, showkatrmir@gmail.com

*Correspondence:
Perwez Alam; aperwez@ksu.edu.sa (+966-551362901, PA).
Mohamed Fahad Alajmi; malajmii@ksu.edu.sa (+966-1-46-77248, MFA).

Table S1: R_f, Linear regression data for the calibration curve of parthenolide (n=6)

**Parameters**	**Parthenolide**
Linearity range (ng/band)	100-700
Regression equation	Y=9.126X + 936.07
Correlation (r^2) coefficient	0.9928
Slope ± SD	9.126 ± 0.08
Intercept ± SD	936.07 ± 25.76
Standard error of slope	0.032
Standard error of intercept	10.51
R_f	0.16 ± 0.001
LOD (ng)	28.46
LOQ (ng)	86.24

Table S2. Recovery as accuracy studies of the proposed HPTLC Method (n=6)

Percent (%) of Parthenolide added to analyte	Theoretical concentration of Parthenolide (ng/band)	Concentration found (ng/band) ± SD	%RSD	% Recovery
0	200	196.09 ± 3.73	1.902	98.04
50	300	296.19 ± 5.39	1.819	98.73
100	400	395.95 ± 6.87	1.735	98.99
150	500	488.20 ± 7.43	1.522	97.64

Table S3. Precision of the proposed HPTLC Method (n=6)

Conc. of standard added (ng/band)	Parthenolide			
	Intra-day Precision		Inter-day Precision	
	Average Conc. found ± SD	%RSD	Average Conc. found ± SD	%RSD
300	294.01 ± 5.33	1.81	292.91 ± 5.01	1.71
400	393.32 ± 6.98	1.77	390.03 ± 6.47	1.65
500	493.46 ± 7.57	1.53	489.29 ± 7.19	1.46

Table 4. Robustness of the proposed HPTLC Method (n=6)

**Optimization condition**	**Parthenolide (300 ng/band)**	
	**SD**	**% RSD**
**Mobile phase composition; (hexane: ethyl acetate)**		
(3:1)	5.11	1.79
(2.8:1.2)	5.19	1.82
(3.2:0.8)	5.23	1.84
**Mobile phase volume (for saturation)**		
(18 mL)	5.29	1.85
(20 mL)	5.18	1.81
(22 mL)	5.37	1.89
**Duration of saturation**		
(10 min)	5.61	1.93
(20 min)	5.53	1.90
(30 min)	5.67	1.95
